# Rapid genome wide mapping of phosphine resistance loci by a simple regional averaging analysis in the red flour beetle, *Tribolium castaneum*

**DOI:** 10.1186/1471-2164-14-650

**Published:** 2013-09-24

**Authors:** Rajeswaran Jagadeesan, Amelia Fotheringham, Paul R Ebert, David I Schlipalius

**Affiliations:** 1 School of Biological Sciences, University of Queensland, St. Lucia, QLD, 4072, Australia; 2 Agri-Science Queensland, Department of Agriculture, Fisheries and Forestry (DAFF), Ecosciences Precinct, Level 3C-West, GPO Box 267, Brisbane, QLD 4001, Australia; 3 Plant Biosecurity Cooperative Research Centre (PBCRC), LPO Box 5012, Bruce, ACT 2617, Australia

**Keywords:** Insecticide resistance, *Rhyzopertha dominica*, Linkage disequilibrium, Fitness cost, Gene interactions

## Abstract

**Background:**

Next-generation sequencing technology is an important tool for the rapid, genome-wide identification of genetic variations. However, it is difficult to resolve the ‘signal’ of variations of interest and the ‘noise’ of stochastic sequencing and bioinformatic errors in the large datasets that are generated. We report a simple approach to identify regional linkage to a trait that requires only two pools of DNA to be sequenced from progeny of a defined genetic cross (i.e. bulk segregant analysis) at low coverage (<10×) and without parentage assignment of individual SNPs. The analysis relies on regional averaging of pooled SNP frequencies to rapidly scan polymorphisms across the genome for differential regional homozygosity, which is then displayed graphically.

**Results:**

Progeny from defined genetic crosses of *Tribolium castaneum* (F_4_ and F_19_) segregating for the phosphine resistance trait were exposed to phosphine to select for the resistance trait while the remainders were left unexposed. Next generation sequencing was then carried out on the genomic DNA from each pool of selected and unselected insects from each generation. The reads were mapped against the annotated *T. castaneum* genome from NCBI (v3.0) and analysed for SNP variations. Since it is difficult to accurately call individual SNP frequencies when the depth of sequence coverage is low, variant frequencies were averaged across larger regions. Results from regional SNP frequency averaging identified two loci, tc_*rph1* on chromosome 8 and tc_*rph2* on chromosome 9, which together are responsible for high level resistance. Identification of the two loci was possible with only 5-7× average coverage of the genome per dataset. These loci were subsequently confirmed by direct SNP marker analysis and fine-scale mapping. Individually, homozygosity of tc_*rph1* or tc_*rph2* results in only weak resistance to phosphine (estimated at up to 1.5-2.5× and 3-5× respectively), whereas in combination they interact synergistically to provide a high-level resistance >200×. The tc_*rph2* resistance allele resulted in a significant fitness cost relative to the wild type allele in unselected beetles over eighteen generations.

**Conclusion:**

We have validated the technique of linkage mapping by low-coverage sequencing of progeny from a simple genetic cross. The approach relied on regional averaging of SNP frequencies and was used to successfully identify candidate gene loci for phosphine resistance in *T. castaneum*. This is a relatively simple and rapid approach to identifying genomic regions associated with traits in defined genetic crosses that does not require any specialised statistical analysis.

## Background

High-throughput “next-generation” sequencing is now a cost effective tool for most genetics laboratories, making new approaches for genetic analysis a possibility. One of the promising aspects of next-generation sequencing data is that it can be used to rapidly define genomic regions of interest in linkage and quantitative genetics experiments. There are myriad ways that this can be accomplished but no straightforward statistical and data processing approach has been developed to distinguish the ‘signal’ from the ‘noise’. Some researchers have simplified the analysis by establishing specially prepared genetic strains, as in linkage by introgression [1]. In other systems sequencing of the parental genomes has been used to allow parentage to be assigned to single nucleotide polymorphisms (SNPs), which is an alternative strategy to facilitate identification of linkage to a trait or traits. It is challenging, however, to confidently determine the parentage assignment of SNPs in genome-wide datasets that have a relatively low average coverage (<10×), such as might be encountered in organisms with large genomes.

In this study we developed a relatively simple and robust method that does not require the parental genomes to be known or sequenced. The method involves bulk-segregant sequence analysis and averaging frequencies of SNPs attaining differential homozygosity to display genomic regions containing genes for a specific trait. We used this method to identify genes conferring resistance to an insecticidal fumigant, phosphine (PH_3_) in a cosmopolitan grain insect pest, *Tribolium castaneum*. Phosphine is an effective fumigant, used worldwide to protect grain harvest from insect pests [2]. It has been in use for nearly five decades owing to its broad spectrum of activity and residue-free characteristics [3,4]. Heavy reliance on phosphine has given rise to high-level heritable resistance in several grain insect pests, threatening the efficacy of phosphine [5-10].

Classical genetic studies on resistance to phosphine in two major grain insect pests, the rust red flour beetle, *T. castaneum*[11] and the lesser grain borer, *Rhyzopertha dominica*[12] has confirmed that strong resistance is primarily conferred by two major genes, *rph1* and *rph2.* Recently, a gene encoding a core metabolic enzyme, dihydrolipoamide dehydrogenase (*dld*) has been identified as *rph2*[13] in both *R. dominica* and *T. castaneum* and a free soil living nematode *Caenorhabditis elegans*, indicating that resistance mechanisms involve altered oxidative metabolism with key respiratory enzymes. *T. castaneum* is a model organism for both genetic and developmental studies and the wealth of background genetic information with a recently published draft genome [14], makes this insect an ideal system in which to test novel applications of next-generation sequencing to gene mapping. The present study aimed to (i) apply next generation sequencing methods to linkage mapping analyses by identifying the genomic location of genes conferring resistance to phosphine and (ii) develop SNP markers for understanding the biological and genetic aspects (gene interactions and fitness) of this trait on grain insect pests.

## Results

### Sequence analyses and SNP discovery

**Figure 1:**
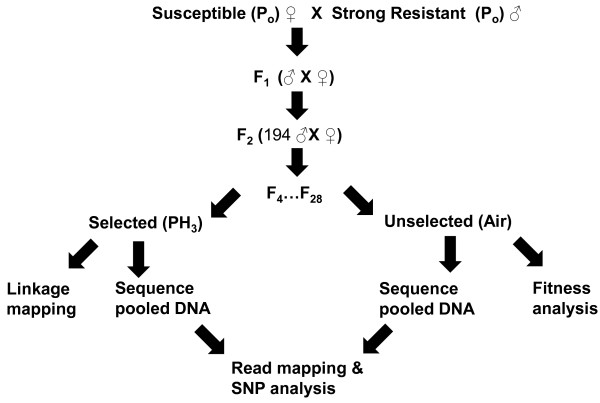
Outline of the crossing scheme and sequencing strategy.

Two similar single pair inter-strain crosses (SIC), SIC_S/SRA_ and SIC_S/SRB_ (both S ♀ X Strong-R ♂) were established (Figure 1) and selected at different generational cohorts (F_4_ and F_19_ for the different crosses) for strong resistance. Selected (strongly resistant) and unselected progeny of these crosses were sequenced using the Illumina GAIIx platform and reads were mapped back to the *T. castaneum* genome assembly (NCBI version 3.0). The outline of the crossing schemes, selections, read mapping and SNP calling criteria are described in more detail in Materials and Methods section. The Illumina sequencing results for the generational selected and unselected pools are outlined in Table 1.

**Table 1:** Results summary of sequencing and read mapping

**Cross**	**Cohort/pool**	**Read length(bp)**	**No. reads**	**Matched reads**	**Total bases matched**	**Approx. coverage**	**No. SNPs called**
SIC_S/SRB_	F_4_ Unselected	36 (paired)	49,636,554	28,119,799	1,012,312,764	5×	556,683
SIC_S/SRB_	F_4_ Selected	36 (paired)	52,622,706	28,155,197	1,013,587,092	5×	297,483
SIC_S/SRA_	F_19_ Unselected	75 (unpaired)	7,944,144	6,443,622	483,271,650	2.4×	310,852
SIC_S/SRA_	F_19_ Selected	75 (paired)	15,643,072	12,246,096	918,457,200	4.5×	602,068

### Identification of candidate regions

**Figure 2:**
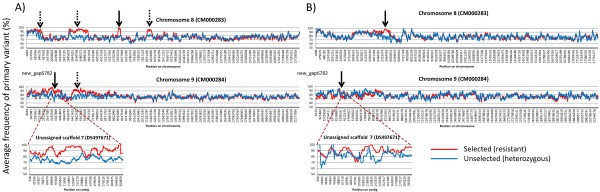
**Average SNP frequency across Chr8 Chr9 and Uns-7, from A) F**_**4 **_**and B) F**_**19 **_**datasets (Selected vs Unselected).** The window size used was an average of 300 cells (or 50 cells for Uns-7) in the spreadsheet analysis. The x-axis is a non-linear scale determined by the number of SNPs in the dataset. The regions showing linkage to resistance are highlighted by a dashed arrow; solid arrow indicates regions where the resistance loci were confirmed by fine-scale linkage mapping.

In order to visualise regions linked to resistance, i.e. selected for homozygosity in phosphine exposed animals, we used a simple spreadsheet analysis of SNP output data from CLC Genomics Workbench. CLC Genomics Workbench outputs each SNP position, the primary variant for each position and the frequency of the primary variant, i.e. the ratio of the number of reads of the variant/total number of matching reads expressed as a percentage. For each generation’s (F_4_ and F_19_) datasets, we combined the two SNP output tables (selected and unselected) for each chromosome into the same spreadsheet and put the primary variant frequencies in separate columns for each dataset. The data tables were then sorted by SNP position, so that the two datasets were aligned in order along the chromosome/scaffold and then the primary variant frequencies were averaged over 300 cells in the spreadsheet for the selected and the unselected columns (Additional file 1: Table S1). The window size does not need to be fixed at 300, but 300 cells gave us the best resolution on these datasets, with the least amount of artefacts caused by low coverage. The averages were then plotted graphically (Figure 2). For the smaller, unassigned scaffolds, we averaged 50 cells or less, depending on how many SNPs were called on each scaffold.

In this way, each plotted data point is an average of a virtual ‘dynamic window’, which changes in size on the corresponding chromosome sequence depending on the number of SNPs called. However the window at any point always covers the same region for both selected and unselected sets and moves down by one SNP each time. The actual physical distance which the dynamic window covers is determined by the span encompassed by the 300 contiguous SNPs in the window. This averaging causes artefacts due to very low SNP density, such as gaps in the scaffold sequence, or high SNP density such as SNP ‘clusters’, to be smoothed out by the analysis. The ideal window size cannot be calculated a priori, and is chosen empirically to reveal candidate regions which should be validated by detailed genetic analysis. The window size does not necessarily change with number of SNPs called, genome size or variant frequency. However, the length of the contig in fragmented genomes will affect the total number of SNPs called for a given contig, so smaller window sizes are preferable for shorter sequences. One consideration though, is that smaller window sizes are affected by the depth of coverage and can show up artefact differences between datasets that are caused by relatively low coverage. The depth of coverage can have large effects on individual SNP frequencies and sometimes regions can have very low coverage which can produce an average of ‘zero’ or be appear to be homozygous in smaller windows. Larger window sizes tend to ‘smooth out’ the analysis and account for SNPs that are missed. In general, there is an inverse relationship with the depth of sequencing coverage and the effective window size that will give reasonable resolution, i.e. the higher the depth of coverage, the better the estimation of SNP frequency will be for any particular SNP, and so the smaller the allowable window size.

The simple averaging method for genome wide analysis of SNP allele frequencies shows major regions linked to phosphine resistance displayed as differential SNP frequency caused by local regions of increased homozygosity in the selected dataset. The method does this without the aid of parentage assignment or other complex statistical calculation of association to the trait. The validity of the method is demonstrated by the fact that the regions found linked to resistance were confirmed in independent crosses from the same parental strains.

In the F_4_ data, four regions were found on Chr8, (region I: 274 to 896 Kbp, region II: 2.7 to 3.8 Mbp, region III: 5.83 to 6.01 Mbp and region IV: 7.96 to 8.54 Mbp), two regions in Chr9 (region I: 2.2 to 4.16 Mbp, region II: 5.07 to 7.2 Mbp) and one region in unassigned genomic scaffold 7 (DS497671), hereafter referred to as Uns-7, (86 to 200 Kbp), that constituted candidate regions associated with phosphine resistance in the *T. castaneum* genome (Figure 2). Analysis of the F_19_ data revealed that on Chr8, only one of the four previously identified regions was nearly homozygous and therefore confirmed to be linked to resistance (Figure 2). In the case of Chr9, neither candidate region previously identified in the F_4_ data was confirmed in the F_19_ analysis, i.e. no increase in the frequency of homozygosity of SNPs was observed in either genomic region in animals that survived exposure to phosphine (Figure 2). However, a region of the previously identified contig Uns-7 was retained as a candidate resistance locus (Figure 2). As a result, subsequent analysis was focused on region III in Chr8 and the candidate region in Uns-7. No other region of the genome showed an appreciable skew toward homozygosity of SNPs in selected versus unselected datasets of the F_19_ generation, indicating that no other resistance loci were present other than the locus on Chr8 and the locus on Uns-7 (Additional file 2: Figure S1).

### Fine scale mapping

A subset of identified SNPs from Chr8 and Uns-7 that were homozygous in selected and heterozygous in unselected F_19_ datasets was chosen for further analysis (Additional file 3: Table S2). In addition, some Chr9 SNPs were included to determine whether the Uns-7 contig mapped to the candidate region of Chr9 that had been identified previously in the F_4_ generation. These SNPs were selected based on the availability of restriction enzymes that could digest the sequence of one allele, but not the other. The local regions around these SNPs were amplified and tested for polymorphism using the appropriate restriction enzymes (i.e. CAPS analysis). In one case there was an informative size polymorphism found after amplification and so was included in the analysis. These markers were analysed in the P_0_ parents of the SIC_S/SRA_ cross, the F_1_ pair used to establish the genetically segregating line, 96 individuals from the F_19_ generation that survived exposure to 0.8 mg L^-1^ phosphine and 160 individuals from the F_28_ generation that survived exposure to 0.5 mg L^-1^ phosphine. The naming convention used in this study describes tc as an acronym for *T. castaneum*, followed by the type of marker used (p) for a PCR-based size polymorphism or c for CAPS followed by their approximate genomic location in kilo (k) or mega (m) bases.

For Chr8, we also tested the linkage to resistance of three of the four identified regions, in 48 selected strongly resistant F_4_ individuals of the cross SIC_S/SRB_ that had survived at a high dose of 0.8 mg L^-1^. Linkage analysis of markers tcp8-777 k (Region I), tcc8-5.975 m (region III) and tcp8-8.11 m (region IV) on selected strongly resistant F_4_ individuals showed that they exhibited >75% linkage to resistance. The marker tcp8-777 k had 9 recombinants, while the marker tcp8-8.11 m had 7 recombinants, corresponding to estimated genetic distances of 3.1 cM and 2.5 cM from the resistance locus respectively. However, only one locus, tcc8-5.975 m, was absolutely linked to resistance in the F_4_ SIC_S/SRB_ cross and was the only one of the four regions that was identified in the F_19_ sequencing and confirmed further in fine scale mapping. This led us to conclude that there was only one locus on Chr8, in region III, and that we had not identified multiple resistance loci on Chr8 that were subsequently lost from the population between F_4_ and F_19_, either due to drift or pleiotropic effects on fitness. It also shows that the low coverage of sequencing identifies linked regions across several cM in the F_4_.

**Figure 3:**
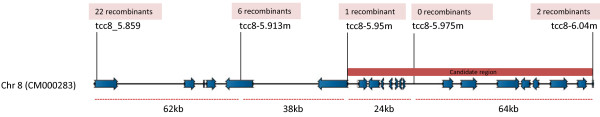
**Fine-scale mapping of Chr8 defining the candidate region *tc*_*rph1.*** The numbers of recombinants for each marker across the region are highlighted in pink, with the defined region containing resistance locus *tc_rph1* in red.

**Figure 4:**
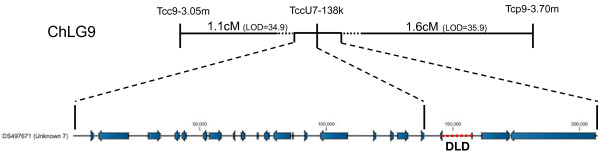
**Linkage mapping of unassigned scaffold (Uns)** - **7 postioning it on chromosome 9.** The region highlighted in red on Uns-7 contains the known DLD resistance locus (tc_*rph2*).

For mapping across the tightly linked Chr8 candidate region identified in the F_19_, SNP markers were designed across the entire candidate region, (Additional file 3: Table S2) and tested in 160 strongly resistant F_28_ individuals. Individuals that were non-recombinant for these SNP markers across the tc_*rph1* candidate region were excluded from the analysis. The results of fine scale mapping defined a candidate region for tc_*rph1* from tcc8-5.975 m to tcc8-6.04 m (Figure 3). Similarly, the marker tccU7-138.2 k with no recombinants in 83 strongly resistant selected F_19_ individuals confirmed the close linkage to second resistance locus, tc_*rph2,* on Uns-7 (Figure 4).

### Positioning the unassigned scaffold (Uns) - 7 into chromosome 9

The loss of homozygosity seen on Chr9 in the F_19_ dataset, compared to the observed homozygous peaks in F_4_ datasets, suggested that an error in genome sequence assembly had resulted in the omission of a segment of DNA sequence from Chr9 and this indicated that a resistance locus did indeed map to Chr9. In such a situation, the finer scale of the F_19_ genetic map would result in the loss of apparent linkage to Chr9. The fact that Uns-7 did contain a resistance locus and was not assigned to any particular site in the genome indicated that it could actually map to the region of Chr9 originally identified as a resistance locus in Chr9. To test this hypothesis, we mapped the relative genetic positions of three SNP markers: tcc9-3.05 m, tcp9-3.70 m (both from region I of Chr9) and tccU7-138.2 k, the marker for tc_*rph2* locus (from Uns-7) in 92 individuals of an F_2_ population of the SIC_S/SRA_ cross (Figure 1). We found that marker tccU7-138.2 k is flanked by tcc9-3.05 m and tcp9-3.70 m, with map distances of 1.1 cM (LOD = 34.9) and 1.6 cM (LOD = 35.9), respectively, confirming our hypothesis that Uns-7 is a part of Chr9 (Figure 4), and most likely positioned in a gap of unknown size between two scaffolds (NW_001093536.1 and NW_001093382.1), designated ‘new_gap6782’ (Figure 2).

### Candidate genes for phosphine resistance

The technique identified a target locus on the Uns-7 scaffold, even though it was not assembled into the larger genome and was itself incomplete and had many gaps in the sequence. This scaffold contains 19 predicted genes, of which two (TC006822 and TC006823) are actually the first and last exons of the dihydrolipoamide dehydrogenase (*dld*) gene (Table 2 and Figure 4). We used our paired end sequencing data to *de novo* assemble the region containing DLD, so that all the exons were represented in the genomic sequence. This assembled contig has been deposited into Genbank (KF032715). Comparative genetic analysis has confirmed this gene as the phosphine resistance locus *rph2* in *T. castaneum*, *R. dominica* and the nematode, *Caenorhabditis elegans*[13]. This was taken as a validation of the SNP averaging technique.

**Table 2:** List of candidate genes for phosphine resistance in Chr8 and Chr9 (Uns-7)

**Gene ID**	**Protein accession**	**Homology in Drosophila**	**E-value**	**Predicted biological function/identity**
**Chr8**				
TC005925	EFA09201	CG5255	7.00E-28	Trypsin like serine protease H65
TC005926	EFA08291	NA	NA	Unknown function
TC005927	EFA08292	CG13804	3.00E-150	Yellow-g2
TC005928	EFA08293	CG6543	3.00E-158	Cyclohex-1-ene-1-carboxyl-CoA hydratase; enoyl CoA hydratase
TC005929	EFA08294	CG2663	3.00E-36	Sec14p-like lipid-binding domain; Alpha tocopherol transfer protein
TC005930	EFA08295	CG13096	1.00E-28	Ribosomal protein L1
TC005931	EFA08296	NA	NA	Unknown function
TC005932	EFA08297	CG5670	0	Na(+)/K(+) ATPase alpha subunit
TC006224	EFA08569	CG12880	5.00E-29	Unknown function
TC006225	EFA08570	CG7463	1.00E-06	Yellow-k
TC006226	EFA08571	CG5717	3.00E-160	Yellow-g
TC006227	EFA08572	CG9792	1.00E-116	Yellow-e
TC006228	EFA08573	NA		Unknown function
TC006229	EFA08574	CG9891	9.00E-89	Yellow-e3
TC006230	EFA08575	CG1629	2.00E-132	Yellow-h
TC006231	EFA08576	CG13279	9.00E-104	Cytochrome b5 related; Fatty acid desaturase
TC006232	EFA08577	CG13279	1.00E-117	Cytochrome b5 related; Fatty acid desaturase
**Chr9 (Uns-7)**				
TC006821	EFA13109	CG17514	0	Translational activator gcn-1, partial; Translator activator activity
TC006822	EFA13110	CG7430	1.00E-48	Dihydrolipoamide dehydrogenase E3 subunit
TC006823	EFA13111	CG7430	1.00E-82	Dihydrolipoamide dehydrogenase E3 subunit
TC006824	EFA13130	CG42698	7.00E-111	Pou domain motif 3
TC006825	EFA13112	CG6551	3.00E-104	Serine/threonine protein kinase
TC006826	EFA13113	CG6551	8.00E-38	Serine/threonine protein kinase
TC006827	EFA13114	CG6551	6.00E-38	Serine/threonine protein kinase
TC006828	EFA13115	CG6178	5.00E-87	Fatty acetyl coA synthese activity
TC006829	NA	CG11715	4.37E-08	Cytochrome P450; Cyp4g15
TC006830	EFA13116	CG3800	1.00E-03	Polyprotein; Nucleic acid binding protein
TC006831	EFA13117	CG14660	3.90E-02	Putative serine protease; Labial associated factor
TC006832	EFA13118	CG3104	1.00E-18	Dopamine transporter; Ankyrin repeats involving in protein-protein interactions
TC006833	EFA13119	CG6178	5.00E-102	AMP binding enzyme; Fatty aceyl coA synthese activity
TC006834	EFA13120	CG6551	6.00E-17	Serine/threonine protein kinase; ATP Binding
TC006835	EFA13121	CG3104	2.00E-18	Dopamine transporter; Ankyrin repeats involving in protein-protein interactions
TC006836	EFA13122	NA	NA	Unknown function
TC006837	EFA13123	CG42231	3.00E-10	Unknown function; Uncharacterized conserved protein
TC006838	EFA13124	CG5245	6.25E-49	Nucleic acid/Zinc ion binding
TC006840	EFA13125	CG14938	3.00E-56	Nucleic acid/Zinc ion binding

The candidate region defined by markers tcc8-5.95 m and tcc8-6.04 m on Chr8 contains 17 gene predictions (Table 2 and Figure 3). While some of these genes have strong homologies to genes of known function, the functions of most of them are either ill-defined or unknown. Therefore further work is required to identify the resistance gene within this region.

### Epistatic interactions between the two resistance loci

The relative contribution to the resistance phenotype of the two loci tc_*rph1* and tc_*rph2* was estimated by genotyping surviving F_4_ individuals of the cross SIC_S/SRA_ (Figure 1) exposed to a range of phosphine concentrations. Robust quantification of the relative strengths is difficult, but an estimate of the relative strengths of each genotype can be gleaned from Table 3 using the range of values that the LC_99.9_ is likely to fall between. The relative strength of the phenotype for each genotypic combination was SS (tc_*rph1*^*ss*^/tc_*rph2*^*ss*^, 0.02-0.03 mg L^-1^) < *S*R (tc_*rph1*^*rr*^/tc_*rph2*^*ss*^, 0.03-0.05 mg L^-1^) < RS (tc_*rph1*^*ss*^/tc_*rph2*^*rr*^, 0.06-0.1 mg L^-1^) < RR (tc_*rph1*^*rr*^ /tc_*rph2*^*rr*^, >4.0 mg L^-1^). The LC_99.9_ value for the fully sensitive (QTC4) parental strain as calculated previously is 0.02 mg L^-1^[11]. Using this as a reference value we can estimate the individual contribution of tc_*rph1* and tc_*rph2*, as approximately 1.5-2.5× and 3.0-5.0×, respectively. When homozygous for both resistance loci, the relative resistance ratio is >200×, demonstrating a strong synergistic interaction between the two loci.

**Table 3:** **Relative contribution of resistance loci, tc_*rph1 *and tc_*rph2***

**Genotypes**		**Dose of phosphine (mg L**^**-1**^**)**														
tc_*rph1*	tc_*rph2*	US*	0.008	0.01	0.02	0.03	0.05	0.06	0.1	0.2	0.5	0.8	1	2	3	4
*ss*	*ss*	6	1	0	1	0	0	0	0	0	0	0	0	0	0	0
*sr*	*ss*	16	11	6	4	0	0	0	0	0	0	0	0	0	0	0
*rr*	*ss*	15	8	6	15	9	0	0	0	0	0	0	0	0	0	0
*ss*	*sr*	8	4	2	2	0	0	0	0	0	0	0	0	0	0	0
*sr*	*sr*	25	21	29	5	1	0	0	0	0	0	0	0	0	0	0
*rr*	*sr*	18	16	19	31	19	5	12	0	0	0	0	0	0	0	0
*ss*	*rr*	3	0	2	1	1	7	2	0	0	0	0	0	0	0	0
*sr*	*rr*	2	3	3	2	10	9	6	8	1	3	0	0	0	0	0
*rr*	*rr*	0	1	4	7	4	11	3	5	7	7	4	3	3	3	1
**No. analysed**		**93**	**65**	**71**	**68**	**44**	**32**	**24**	**13**	**8**	**10**	**4**	**3**	**4**	**3**	**1**
PCR amplified		96	90	71	68	44	32	28	14	10	10	4	4	4	3	2
Survivors		202	107	155	90	75	40	30	14	11	11	4	6	4	3	3
Total no.of insects bioassayed		202	130	201	201	200	313	204	295	312	300	320	302	310	300	320

*US = Unselected beetles.

### Relative fitness of tc_*rph1* and tc_*rph2*

We estimated the change in frequency of resistance alleles in the absence of phosphine selection over 18 generations, from F_2_ to F_20_, using the markers tcc8-5.975 m (tc_*rph1*) and tccU7-138.2 k (tc_*rph2*) and analysed deviation from Hardy-Weinberg equilibrium using chi-square test (Table 4). The results showed a steady increase in the homozygous-resistant genotype tc_*rph1*^*rr*^ in each generation from F_5_ to F_20_ (***χ***^2^ = 18.8; *P* < 0.001, df = 1), indicating that tc_*rph1* may have a selective fitness advantage associated with phosphine resistance. In contrast, tc_*rph2* showed a significant decrease in resistant homozygotes (tc_*rph2*^*rr*^) coupled with a steady increase in sensitive homozygotes (tc_*rph2*^*ss*^) over 18 generations (***χ***^2^ = 75.2, *P* < 0.001, df = 1) indicating a strong fitness cost associated with this resistance allele in the absence of selection.

**Table 4:** **Fitness analysis of resistance loci, tc_*rph1 *and tc_*rph2 *over 18 generations in the absence of phosphine selection**

**Generations**	**Insects tested**	**Genotypes**			**Allelic frequency**		**Chi-square analysis on genotypes**^**†**^	
		***rr***	***rs***	***ss***	***p allele***	***q allele***	***χ***^**2**^**(df = 1)**	***P *value**
**tc_*rph1***								
F_2_	94	28	41	27	0.51	0.49	2.15	0.143
F_5_	94	34	43	17	0.59	0.41	6.83^*^	0.009
F_10_	96	38	45	13	0.63	0.37	13.4^**^	<0.001
F_15_	95	35	46	14	0.61	0.39	9.38^*^	0.002
F_20_	96	38	48	8	0.65	0.34	18.8^**^	<0.001
**tc_*rph2***								
F_2_	94	20	52	22	0.49	0.51	1.15	0.283
F_5_	96	5	54	37	0.33	0.67	22.8^**^	<0.001
F_10_	92	7	38	45	0.29	0.71	33.6^**^	<0.001
F_15_	96	0	28	64	0.15	0.85	99.0^**^	<0.001
F_20_	96	0	36	58	0.19	0.81	75.2^**^	<0.001

* Significant (*P* < 0.01); ** Significant (*P* < 0.001).

^†^ Significance values are calculated from deviation from Hardy-Weinberg equilibrium compared to expected F_2_ (1:2:1) ratio.

## Discussion

Finding single nucleotide changes that cause large phenotype changes in highly polymorphic genetic backgrounds is a formidable task. In the present study we have taken advantage of next-generation sequencing technology and the published reference genome sequences of *T. castaneum* to rapidly identify the genomic locations of phosphine resistance loci in *T. castaneum*. Our results demonstrate the power of our simple averaging method to scan for local regions of loss of heterozygosity, which is preferable to standard linkage mapping for identifying gene regions responsible for well-defined traits in advanced intercrosses. The method does not rely on a high quality reference genome, high depth of sequence coverage, use of inbred strains or parentage assignment of SNPs. The whole genome including multiple unassigned scaffolds can be easily scanned to identify linked regions.

The simple averaging method also has advantages in that it can reduce artefacts caused by gaps in reference genome assemblies. Large gaps that are included in the assembled sequence, as is the case for *T. castaneum*, can cause major problems for analyses that rely on defined physical distances, as there would appear to be no variants or genes within these gaps. Variable SNP density can also cause problems for analyses that rely on defined physical distances. Our technique eliminates this problem by allowing the SNPs in the datasets to define the region to be averaged and how far the analysis window is shifted along the chromosome for each iteration, rather than a user-defined physical distance. This is easily visualised with spreadsheet software, such as Excel™.

Our analysis defined two genomic regions on Chr8 and Chr9 (Uns-7) containing loci responsible for high-level resistance, tc_*rph1* and tc_*rph2*. The results of F_4_ SNP analysis showed discontinuous SNP frequency signals in the candidate regions of both Chr8 and Chr9, represented as separate peaks in the F_4_ SNP frequency graphs (Figure 2) and suggested the possible disorientation or erroneous order of chromosomal scaffolds in the reference genome, either due to lack of genetic markers or low recombination in these regions in the crosses used to create the linkage map [15]. Our inheritance analysis of selected SNP markers from Chr8, Chr9 and Uns-7 on a F_2_ mapping population confirmed this and identified the location of Uns-7 on Chr9 (new_gap6782). Similar chromosomal sequence discrepancies on the *T. castaneum* reference genome especially on Chr9 were observed by Wang *et al.*[16] and the respective sequences were later rearranged to their proper location.

### Candidate genes

Our next-generation sequencing and mapping results show that the major candidate resistance gene for tc_*rph2* is on Uns-7. This scaffold contains the dihydrolipoamide dehydrogenase (*dld*) gene, which was confirmed as a resistance locus using more traditional methods that required much greater effort and expense [13]. This demonstrates the validity, power and efficiency of the simple averaging method. All sequence scaffolds, including those not assigned to chromosomes can be scanned independently and rapidly for linkage to a trait. Since more than 22 Mb (10%) of the *T. castaneum* genome sequence is still unassigned to genomic locations, this represents a powerful way of detecting linkage, even across smaller regions in fragmented genome assemblies.

At the tc_*rph1* locus, the candidate gene list contains 17 predicted genes. However, none of them are associated with the same pathways as *dld*, consistent with the hypothesis of strongly resistant insects having at least two different mechanisms of resistance to phosphine [2,17-20]. Although it is difficult to discuss the potential roles of each of the candidates, genes in the mapped interval are involved in lipid metabolism, chitin metabolism, membrane permeability and maintaining ion gradients. A resistance gene in any of these pathways would be consistent with previous observations on phosphine mode of action through oxidative stress and lipid peroxidation and resistance through reduced uptake [13,19-24].

### Epistatic interactions between the two resistance loci

We used markers tightly linked to resistance loci tc_*rph1* and tc_*rph2* to determine their relative phenotypic effects and genetic interactions in their homozygous and heterozygous states. In the genotype analysis, we showed that the resistance locus tc_*rph2* exhibited a stronger phenotypic effect compared to tc_*rph1.* Both resistance loci are incompletely recessive but when both in a homozygous state they interact synergistically with resistance levels greater than 200×. This is a conservative estimate based on survival of the *rph1*^rr^/*rph2*^*rr*^ genotype at doses up to 4.0 mg L^-1^, the highest dose tested. These results are in accordance with previous results showing synergistic interactions between resistance genes with resistance levels up to 431×, estimated using LC_50_ response values [11]. The synergism observed between the resistance alleles of *T. castaneum* resembles the synergistic interaction of *rph1* and *rph2* in *R. dominica*[6]. These results are also consistent with the two gene model of high-level phosphine resistance seen in *R. dominica*[6,12,25].

### Pleiotropic effects on fitness

Previous analysis [11] on the relative change in the resistance phenotype in the absence of phosphine selection showed the existence of a possible fitness disadvantage in a *T. castaneum* population segregating for strong resistance alleles. In the present study, we observed a significant decrease in the frequency of the tc_*rph2*^*rr*^ homozygous resistant genotype over 18 generations with a corresponding increase in heterozygote (tc_*rph2*^*rs*^) and susceptible genotypes (tc_*rph2*^*ss*^). This directly shows the presence of significant selective fitness disadvantage for homozygotes at the tc_*rph2* locus. Field population based fitness studies [26-29] on phosphine resistant insect strains of *R. dominca, T. castaneum, Sitophilus oryzae*, *Cryptolestes ferrugineus* and *Oryzaephilus surinamensis* reported the existence of biological fitness cost associated with phosphine resistance.

In contrast to tc_*rph2*, we observed a significant increase in the frequency of the homozygous resistant allele of tc_*rph1*, suggesting a selective fitness advantage of weakly resistant homozygotes (tc_*rph1*^*rr*^) compared to susceptible genotypes. These results are consistent with previous fitness estimates on weakly resistant strains of *T. castaneum, R. dominica* and *S. oryzae*[30] and shows that there are fundamental differences between the genes that are associated with each resistance locus.

## Conclusion

The results of the present study used a whole genome resequencing approach and we validated our method of processing the sequences for rapid scanning of polymorphisms and identifying the candidate genes associated with phosphine resistance trait in *T. castaneum* by linkage mapping*.* Although we have only validated the technique for a strongly selected and previously characterized trait, theoretically it is transferrable to nearly any species that has some genomic or transcriptomic reference data against which reads may be aligned, whose breeding may be manipulated for the generation of specific populations for linkage mapping. The detailed genotype analysis using identified SNP markers demonstrated that high-level resistance in *T. castaneum* is a result of two synergistic genes, tc_*rph1* and tc_*rph2* with the latter having significant fitness cost. The fine-scale mapping analysis also defined the genomic regions containing these loci. The results show the validity of the technique and that it can be applied in cases where parental genotypes are unknown and in circumstances where the genome coverage conferred by sequencing is relatively low, which would often be the case for organisms with large genomes.

## Methods

### Beetle strains

Two strains of *T. castaneum* were used, a phosphine susceptible strain, QTC4 (‘S’ strain) and a strongly resistant strain, QTC931 (Strong-R). The ‘S’ strain was derived from adults collected from a storage facility in Brisbane, southeast Queensland, Australia in 1965 [31] and since then, the population of this strain has been maintained in the laboratory without exposure to phosphine and is fully susceptible to phosphine. The Strong-R strain was derived from adults collected from central storage at Natcha, southeast Queensland, and Australia in 2000 [11]. Homozygosity for resistance was selected within the ‘Strong-R’ strain by phosphine selection for at least five generations. In addition, both S strain and Strong-R exhibit a linear response to phosphine in a probit mortality response analysis, indicating their respective homozygosity for the susceptibility/resistance trait [11]. The insects were cultured in whole wheat flour and yeast 20:1 and maintained at 30°C and 55% relative humidity (RH).

### Genetic crosses

Two similar single pair inter-strain crosses (SIC), SIC_S/SRA_ and SIC_S/SRB_ (both S ♀ X Strong-R ♂) were established (Figure 1) between these strains as described previously [11]. For each cross, single virgin adult male and female siblings of the F_1_ progeny were mated to derive 194 F_2_ individuals, which were allowed to mate freely for several weeks to produce the F_3_ generation. After producing the F_3_ generation, 94 F_2_ individuals were retained and used for linkage mapping and SNP validation. The F_3_ and subsequent progeny were kept as discrete generational cohorts up to the F_28_ (Figure 1), and each further generational cohort consisted of approximately 500 to 1000 beetles. The SIC_S/SRA_ cross was established independently 20 months before the SIC_S/SRB_ cross and aimed to establish genetic map for *T. castaneum*. The latter (SIC_S/SRB_) cross was established for the express purpose of genome resequencing to identify larger candidate regions that could then be fine-scale mapped in the later generational cohorts (F_19_) of the SIC_S/SRA_ cross without having to wait another 16 months for the generations to progress. As parental strains, S and Strong-R of these two crosses were highly homozygous in responding to the resistance trait, these two crosses were considered as a replicates.

### Fumigation

Phosphine gas generation and all fumigation bioassays were performed for 20 hours at 25°C in sealed airtight desiccators as described previously by Daglish *et al.*[32] and Jagadeesan *et al.*[11].

### DNA extraction and sample preparation for sequencing

A high discriminatory dose (0.8 mg L^-1^) selection was performed to isolate individuals that were homozygous for resistance trait by exposing more than 10,000 F_4_ progeny of the cross SIC_S/SRB_ and 20,000 F_19_ progeny of the cross SIC_S/SRA_ (Figure 1). For each cross, total genomic DNA was extracted from 100 survivors of each selection and 100 unselected beetles. The DNA was extracted using a QIAGEN DNeasy extraction kit according to the manufacturer’s protocol. The F_4_ samples were sequenced using the Illumina GAIIx platform with paired-end reads of 35 bp and the F_19_ was subsequently sequenced with 75 bp (paired end for selected, unpaired for unselected) (Figure 1).

### DNA extraction and sample preparation for SNP genotyping and mapping

A modified high-salt DNA extraction procedure [33] was adopted for genotyping individual insects. Genomic DNA was extracted from the P_0_ parents, F_1_ hybrids, F_2,_ F_4_, F_19_ and F_28_ individuals of the SIC_S/SRA_ intercross for fine-scale mapping of the resistance locus. Briefly, individual insects were placed in a 96-well PCR microtitre plate, snap frozen in liquid nitrogen and homogenized in 100 μL lysis buffer (0.4 M NaCl, 10 mM Tris–HCl pH 8.0 and 2 mM EDTA pH 8.0) using a multi pin header, for 10-15 s. Then 4 μL of 10% SDS (4% final concentration) and 1 μL of 10 mg ml^-1^ proteinase K (100 μg ml^-1^ final concentration) were added to each sample and mixed well. The samples were incubated at 37°C for overnight, after which 25 μL of 6 M NaCl (NaCl saturated H_2_O) was added to each. Samples were vortexed for 30 s at maximum speed, and tubes centrifuged for 45 min at 1500 *g*. The supernatant was transferred to fresh tubes. To each sample, an equal volume of ice cold 100% ethanol was added, mixed well, and then centrifuged for 45 min, at 1500 *g*. The pellet was washed with 70% ethanol, air dried and resuspended in 25 μL sterile distilled H_2_O. Finally, the samples were kept at 65°C for 10 min to denature any residual DNAses. The genomic DNA template was subsequently quantified and diluted to 2 ng μL^-1^ before inclusion in PCR.

### Mapping short reads to the *T. castaneum* reference genome

The selected and unselected re-sequenced data sets from F_4_ and F_19_ progeny of the crosses SIC_S/SRB_ and SIC_S/SRA_ (Figure 1) were mapped separately to the reference *T. castaneum* genome (version Tcas_3.0, from NCBI) using CLCBio Genomics Workbench v4.9 software [34]. The F_4_ and F_19_ sequence data sets were mapped to the reference under the read mapping algorithm. Read mapping was performed with high stringency, requiring a perfect match and no unaligned ends with the following default parameters, Match +1 Mismatch − 2; Deletion − 3 and Insertion − 3.

### SNP detection

Quality-based SNP detection in CLC Genomics Workbench is based on the Neighbourhood Quality Standard (NQS) algorithm described by [35,36]. For a SNP to be called, the default quality specifications such as window length (11), maximum number of gaps and mismatches (2), minimum average quality of surrounding bases (15) and minimum quality of the central base (20) were chosen together with a minimum coverage of six and minimum variant frequency of 35% within a pool.

The SNP detection output tables for selected and unselected F_4_ and F_19_ datasets obtained were directly exported to a separate Excel™ spreadsheet. SNP datasets for selected (resistant) and unselected (segregating) progeny were compared by separating the data by generation and chromosome, and sorted according to the reference base position and with the frequency of the primary variant, i.e. the most common allele for a SNP regardless of reference base identity, for the selected and unselected datasets being in separate columns. The frequency of the primary variant was then averaged over 300 cells in the spreadsheet for each dataset for chromosomes and 40 cells for unassigned scaffolds, given that the number of SNPs/scaffold is greatly reduced due to their smaller sizes.

### SNP validation and analysis

A subset of SNPs were selected from identified candidate gene regions for further investigation from regions where the averaged SNP allelic frequency attained near homozygosity (90-100%) in selected datasets but remained heterozygous (~50-80%) in the unselected datasets (Additional file 3: Table S2 and Figure 1). SNPs of interest that were identified to be on restriction enzyme (RE) recognition sites, and thus amenable to CAPS analysis, were amplified from individual insects by PCR. For each insect, 4 ng of template DNA was amplified in a reaction volume of 25 μL containing: 1× PCR buffer, 4 mM MgCl_2_, 0.8 mM dNTPs (New England Biolabs), 1.25 U μl^-1^ Taq DNA polymerase (Bioline), 0.4 μM specific forward and reverse primers. The PCR conditions were: denaturation for 2 min at 95°C; then 40 cycles of 95°C for 45 s, 52°C for 45 s, 72°C for 45 s with a final extension at 72°C for 2 min. Restriction digestions were carried out in 15 μL final volume containing 5 μL of the PCR product, 1× concentrated restriction enzyme buffer with optional 1× Bovine Serum Albumin (BSA), and 1-3 U of RE for at least one hour to overnight at the approriate temperature. Products were analysed by 2.5% agarose gel electrophoresis (1× TAE, 200 V for 30 min).

### Mapping of candidate regions

The CAPS markers from candidate regions in Chr8 (Genbank accession CM000283), Chr9 (CM000284) and Uns-7 (DS497671) were genotyped in parents and F_1_ for polymorphism and tested for Mendalian segregation in the segregating F_2_. The F_2_ genotypes were analysed by Map Manager QTXb20 and tested for 1:2:1 segregation of parental alleles using *G* test at 95% confidence interval [37,38]. Marker order was determined with a LOD score threshold of 3.0 and map distances were estimated by the Kosambi function [39].

The fine scale mapping was carried out on markers linked to resistance loci tc*_rph1* and tc_*rph2* to estimate the distance between each marker and their respective target genes. This was achieved by genotyping the segregation profile of markers linked to tc_*rph1* and tc_*rph2* in survivors of F_4_ (SIC_S/SRB_), F_19_ (SIC_S/SRA_) selected at high discriminating dose of 0.8 mg L^-1^ and F_28_ (SIC_S/SRA_) progeny at 0.5 mg L^-1^, respectively (Figure 1). We reduced the discriminatory dose from 0.8 to 0.5 mg L^-1^ for the selection at F_28_ progenies, owing to the relatively small size of population of 4700 beetles produced in this generation and moreover, this will not affect selecting strongly resistant homozygous insects [11]. Surviving individuals, not homozygous for the resistance alleles (tc_*rph1* and tc_*rph2*) were scored as recombinant at that locus. Individuals who were heterozygous across the candidate region were excluded from the analysis.

### Determination of relative contribution and fitness cost of tc_*rph1* and tc_*rph2*

To identify the relative contributions of resistance loci, tc_*rph1* and tc_*rph2*, a selection at series of concentrations of phosphine from 0.008, 0.01, 0.02, 0.03, 0.05, 0.06, 0.1, 0.2, 0.5, 0.8, 1.0, 2.0, 3.0 and 4.0 mg L^-1^, each with approximately 150–200 insects of F_4_ progeny of the cross SIC_S/SRA_ was performed (Figure 1) and the surviving individuals at each concentration were genotyped using the respectively linked SNP markers, tcc8-5.975 m and tccU7-138.2 k. The effect of resistance phenotypes for different allelic combinations was measured by comparing the genotype pattern of these markers over increasing concentrations of phosphine. The same markers were also used to investigate changes in allele frequency associated with resistant (*rr*) and sensitive (*ss*) alleles of both the loci tc_*rph1* and tc_*rph2* in the absence of selection. The beetle populations not exposed to phosphine (unselected) were sampled randomly (~96) at discrete generations, F_5_, F_10_, F_15_ and F_20_ and genotyped for both the resistance loci to determine any observable fitness/heritability effects associated with resistance loci in the absence of selection using a chi-square test.

## Abbreviations

Bp: Base pairs; CAPS: Cleaved Amplified Polymorphic Sequences; Chr: Chromosome; dNTPs: Deoxy Nucleotide Triphosphates; Gbp: Gig base pairs; Kbp: Kilo base pairs; LOD: Logarithmic odd score; Mbp: Mega base pairs; NQS: Neighbourhood Quality Score; PCR: Polymerised Chain Reaction; PH3: Phosphine; RE: Restriction Enzyme; S/SRA: Cross A: Susceptible X Strong-R (A); S/SRB: Cross B: Susceptible X Strong-R (B); SIC: Single pair Inter-strain Cross; SNPs: Single Nucleotide Polymorphisms; TAE: Tris-acetate-EDTA [Ethylenediaminetetraacetic acid]; tc_rph1: Gene1 conferring resistance to phosphine in *Tribolium castaneum*; tc_rph2: Gene2 conferring resistance to phosphine in *Tribolium castaneum*; U: Units; Uns: Unassigned genomic scaffold; V: Volts.

## Competing interests

The authors declare that they have no competing interests.

## Authors’ contributions

RJ, DS and PE designed the study. Bioinformatic analyses were performed by DS with input from RJ. RJ performed the genetic crosses, genome sequencing and linkage mapping experiments and AF contributed to the fine scale mapping. RJ and DS drafted the manuscript; and PE revised the manuscript and provided critical comments. All authors read and approved the final manuscript.

## Supplementary Material

Additional file 1: Table S1 An example table for averaging variant (SNP) frequency for chromosome 8 for the F_4_ generation Selected vs Unselected datasets in an Excel™ spreadsheet. Click here for file

Additional file 2: Figure S1 Illustrating the average SNP frequency across all chromosomes from F_4_ and F_19_ datasets (Selected vs Unselected). Click here for file

Additional file 3: Table S2 Listing markers used for mapping resistance loci on Chr8 and Chr9 (Uns-7). Click here for file
