# Supplementary figures and images for: Rapid genome wide mapping of phosphine resistance loci by a simple regional averaging analysis in the red flour beetle, Tribolium castaneum
Source: BMC Genomics. 2013 Sep 24;14:650. doi: 10.1186/1471-2164-14-650 (PMC3849015; doi:10.1186/1471-2164-14-650)

Average frequency of primary variant (%)

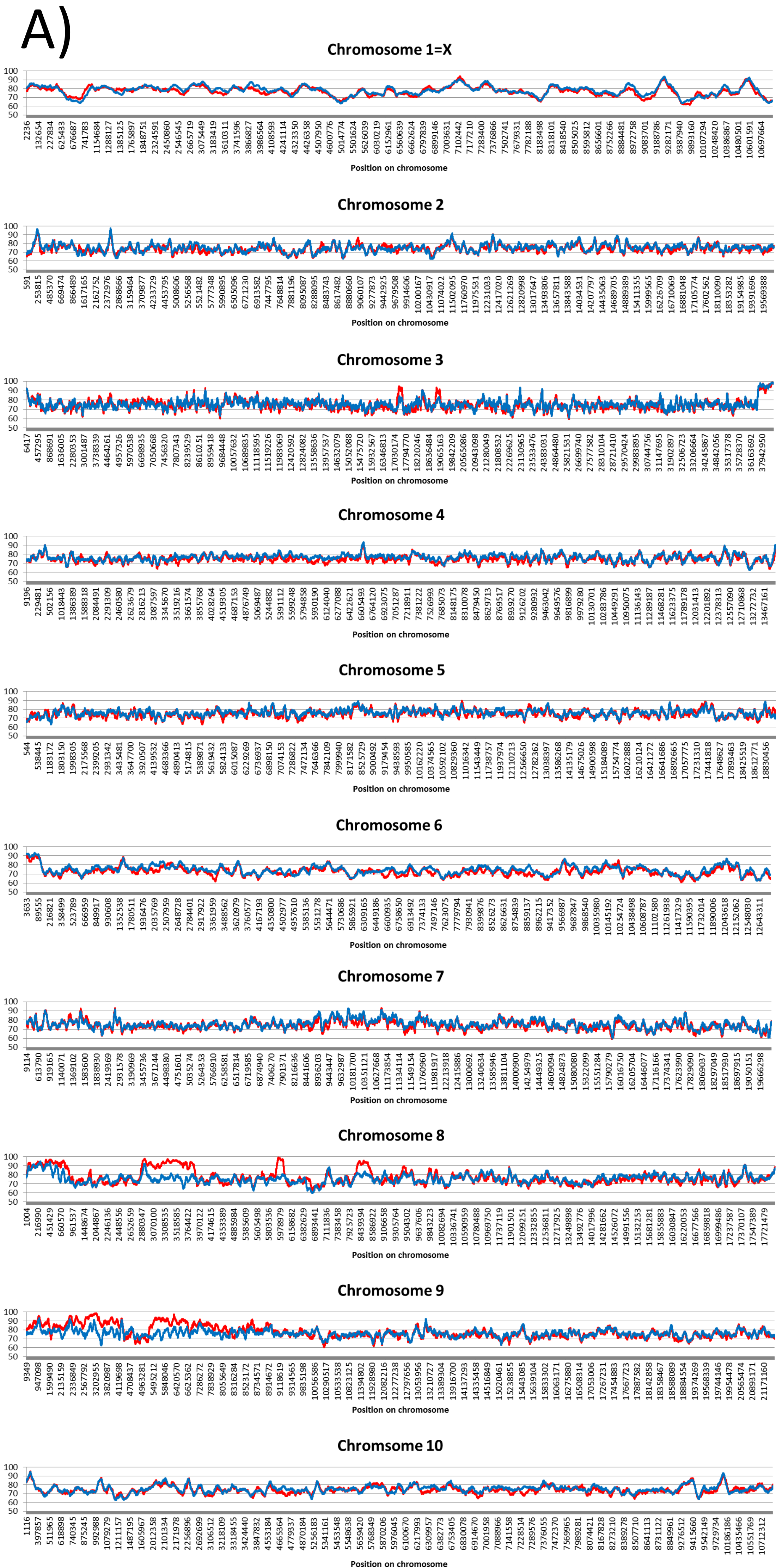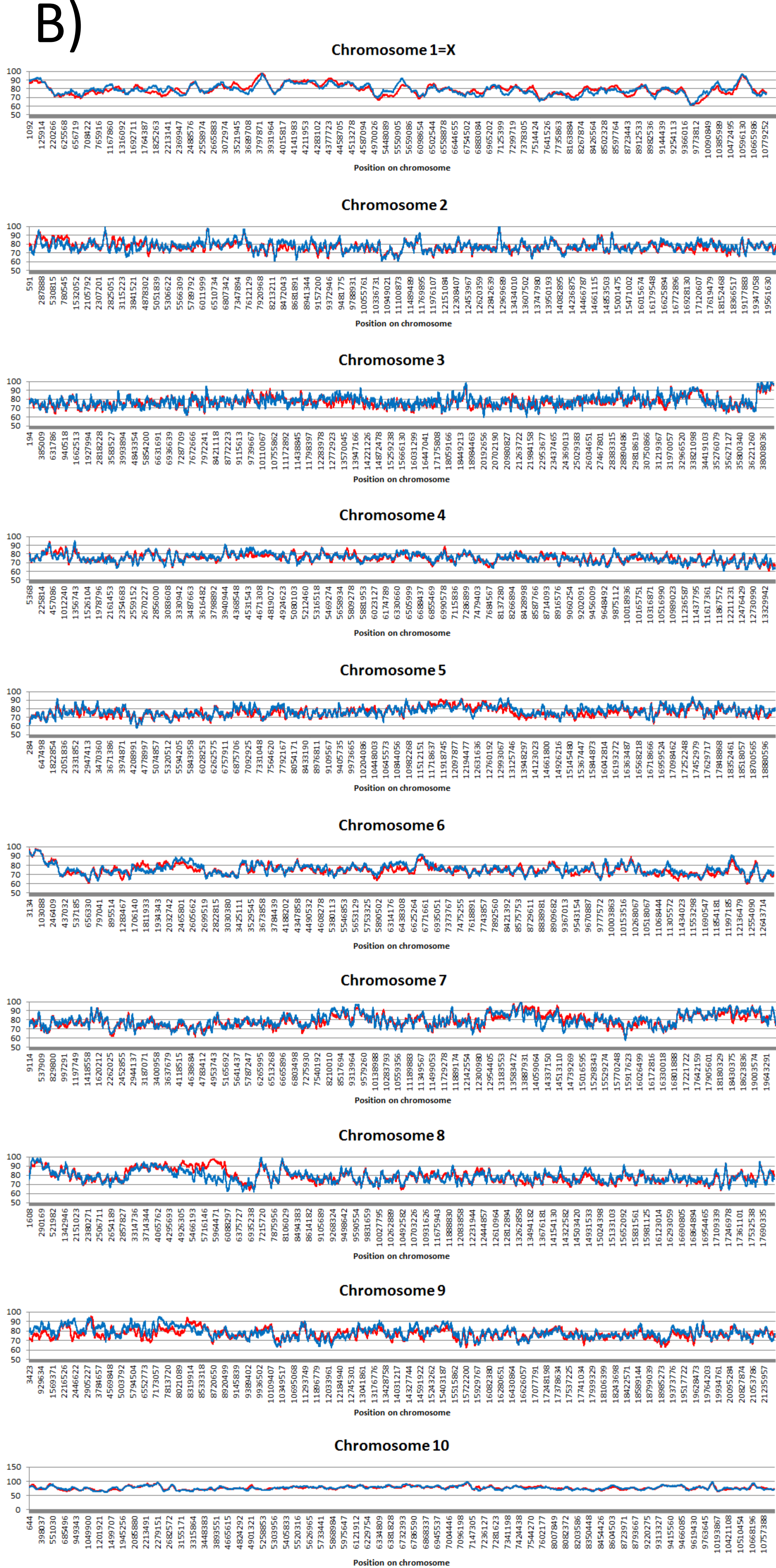

Selected

Unselected

Supplement: Additional file 2: Figure S1 — Illustrating the average SNP frequency across all chromosomes from F4 and F19 datasets (Selected vs Unselected). [file 1471-2164-14-650-S2.pdf]
