# Supplementary material for: Rapid genome wide mapping of phosphine resistance loci by a simple regional averaging analysis in the red flour beetle, Tribolium castaneum
Source: BMC Genomics. 2013 Sep 24;14:650. doi: 10.1186/1471-2164-14-650 (PMC3849015; doi:10.1186/1471-2164-14-650)
Supplement: Additional file 3: Table S2 — Listing markers used for mapping resistance loci on Chr8 and Chr9 (Uns-7). [file 1471-2164-14-650-S3.doc]

**Table S2: List of SNP markers used for scanning resistance polymorphisms in Chr8 and Chr9 (Uns-7)**

| **Marker** | **Accession** | **Forward Primer** | **Reverse Primer** | **Position** | **Reference allele** | **Allele Variants** | **Enzyme** | **Genotype cut by the enzyme** |
| --- | --- | --- | --- | --- | --- | --- | --- | --- |
| **Chr8** |  |  |  |  |  |  |  |  |
| tcp8-77.7k | CM000283 | CCGGGAGACCCACATAAAA | ACCGTCCGATTTTAGTGTCAAT | 776839..777457 | NA | Dominant | NA | NA |
| tcc8-5.859m | CM000283 | CAAGACAACTCGCATCAA | TAATCACCGGACTCGAAA | 5859918 | T | C/T | MnII | Strong-R |
| tcc8-5.913m | CM000283 | GAACATTGACTCGACTGCGG | ATCGTCTCAAACCAGCCTCAA | 5913021 | A | C/A | BsaXI | Susceptible |
| tcc8-5.95m | CM000283 | TGGGCTTGGATTTCGGTTAG | ATTTTCATTCCTCCCCGATTTC | 5951399 | A | G/A | MboII | Susceptible |
| tcc8-5.975m | CM000283 | ACTGAACTGCCTCAACTAAAAC | CGATTTGTTTTTCTGCACGTTC | 5975851 | C | G/C | MspI | Susceptible |
| tcc8-6.04m | CM000283 | TTGAAAAATGGGCTGCAGTGG | CAAAAGGGCCAAACTAACATA | 6040718 | T | C/T | MseI | Susceptible |
| tcp8-8.11m | CM000283 | TGTGATTGTACAGCGTGGTGA | AAACGGGAGATAAACAGAGAGA | 8118003..8118400 | NA | Dominant | NA | NA |
| **Chr9 (Uns-7)** |  |  |  |  |  |  |  |  |
| tcc9-3.05m | CM000284 | CTAAGCCATTCCCTAACACC | CATTTCCGCCAATCTTACTT | 3051916 | A | A/C | MseI | Susceptible |
| tcp9-3.70m | CM000284 | CCGCACATCATTAGAAAGCTTA | GTGTTGTTCATAGTATGGTAGC | 3707346…3708083 | NA | Codominant/ size variant | NA | NA |
| tccU7-138.2k | DS497671 | CTAACTGCACATCAACTAACAC | GAATCGTTTGGTGTGTCTCTA | 138279 | C | T/C | EcoRI | Susceptible |
